# Reciprocal regulation of autism-related genes MeCP2 and PTEN via microRNAs

**DOI:** 10.1038/srep20392

**Published:** 2016-02-04

**Authors:** Jing-Wen Lyu, Bo Yuan, Tian-Lin Cheng, Zi-Long Qiu, Wen-Hao Zhou

**Affiliations:** 1 Departments of Neonatology, Children’s Hospital of Fudan University, Shanghai 201102, China; 2 Institute of Neuroscience, Shanghai Institutes for Biological Sciences, Chinese Academy of Sciences, Shanghai 200031, China; 3 Key Laboratory of Birth Defects, Children’s Hospital, Fudan University, Shanghai 201102, China

## Abstract

*MeCP2* encodes a methyl-CpG-binding protein that plays a critical role in repressing gene expression, mutations of which lead to Rett syndrome and autism. *PTEN* is a critical tumor suppressor gene that is frequently mutated in human cancers and autism spectrum disorders. Various studies have shown that both MeCP2 and PTEN proteins play important roles in brain development. Here we find that MeCP2 and PTEN reciprocally regulate expression of each other via microRNAs. Knockdown of MeCP2 leads to upregulation of microRNA-137, which in turn represses expression of PTEN, thus PTEN would be down-regulated when MeCP2 is knockdown. Furthermore, we find that deletion of PTEN leads to phosphorylation of Serine 133 of CREB, then increases the expression of microRNA-132. miR-132 inhibits the expression of MeCP2 by targeting on the 3′UTR of MeCP2 mRNA. Our work shows that two critical disorders-related gene MeCP2 and PTEN reciprocally regulate expression of each other by distinct mechanisms, suggesting that rare mutations in various disorders may lead to dysregulation of other critical genes and yield unexpected consequences.

MeCP2 belongs to a family of methyl-CpG-binding proteins that regulate gene expression by DNA methylation via recruitment of histone deacetylases^1^,^2^. MeCP2 is indispensable for neural development, for example regulating expression of the brain-derived neurotrophic factor (BDNF) gene^3^. MeCP2 has demonstrated a key role in synaptic homeostatic plasticity^4^,^5^. The *Mecp2*-null mouse is a mouse model of Rett syndrome^6^, a severe form of autism-spectrum disorder^7^.

*PTEN* is a tumor suppressor gene that negatively regulates the phosphatidylinositol-3-kinase (PI3K)/AKT signaling pathway^8^, which in turn plays a critical role in regulating cell growth, survival, and proliferation. Abnormalities in PTEN lead to neurological and psychiatric disorders such as brain tumors, autism, macrocephaly, seizures, mental retardation, and schizophrenia^9^,^10^,^11^.

MicroRNAs (miRNAs) are 20–25-nucleotide-long, noncoding RNAs that modulate gene expression and development by post-transcriptionally targeting RNA-induced silencing complexes^12^. miRNAs have important regulatory functions in basic biological processes such as development, cellular differentiation, proliferation, apoptosis, and tumorigenesis^12^,^13^,^14^. The expression of miRNAs was shown to be altered in the brains of *Mecp2*-null mice^15^,^16^. Furthermore numerous miRNAs have been shown to regulate PTEN expression^17^,^18^,^19^,^20^,^21^,^22^.

In this study, *Mecp2* knockdown repressed PTEN expression and increased AKT phosphorylation. Furthermore, the *Mecp2*-mediated effect on PTEN expression occurs via a mechanism involving miR-137. Interestingly, we also found that MeCP2 expression was down-regulated by PTEN short hairpin RNA. We further found that phosphorylation of Ser-133 of cyclic AMP-response-element-binding-protein (CREB), a substrate of PTEN phosphatase, increased after knocking down PTEN then led to down-regulation of MeCP2 targeted by miR-132. Our work revealed that the two critical genes, *Mecp2* and *Pten,* regulate expression of each other by microRNA targeting and yield further molecular insights for disorders-related mechanisms.

## Results

### PTEN down-regulated by *Mecp2* knockdown

**Figure 1:**
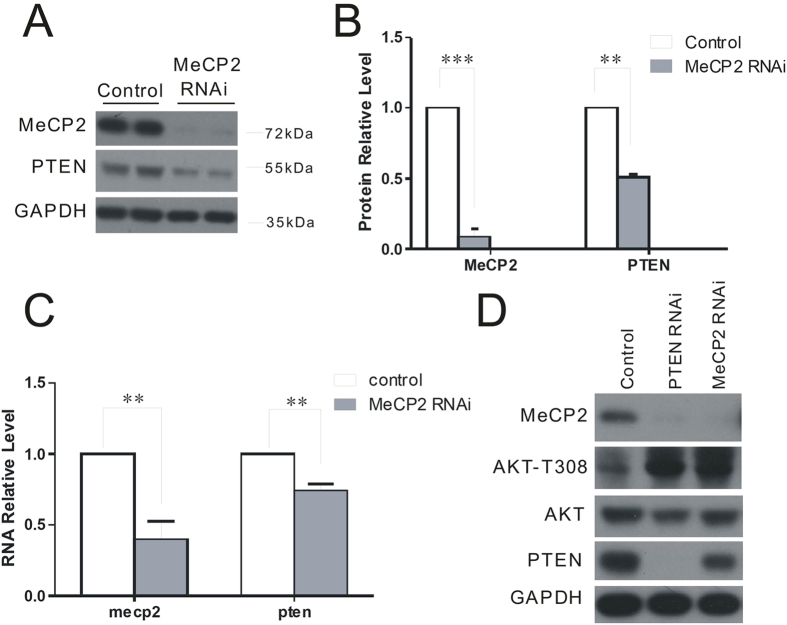
MeCP2 deficiency down-regulates PTEN. (**A)** Primary cultured neurons were transduced with lentivirus expressing GFP (control) or MeCP2 RNAi (for *Mecp2* knockdown). PTEN protein expression was down regulated in MeCP2-RNAi neurons. Glyceraldehyde phosphate dehydrogenase was used as a loading control. **(B)** MeCP2 and PTEN protein levels in (**A**) were quantified using Image J. **(C)** *Mecp2* and *Pten* RNA levels were analyzed by qPCR. **(D)** AKT T308 phosphorylation status was increased by PTEN RNAi or MeCP2 RNAi compared with controls.

To determine if MeCP2 may affect the expression of PTEN, we cultured primary neurons from the mouse brain, and transfected with lentivirus expressing green fluorescent protein (GFP) (control) or short hairpin MeCP2 (for *Mecp2* knockdown). Surprisingly, we found that PTEN protein expression was significantly reduced after 5 days in neurons transfected with MeCP2 RNAi compared with controls (Fig. 1A,B). Consistently, *Pten* mRNA expression was also reduced in neurons with MeCP2 knockdown, as shown by quantitative real-time polymerase chain reaction (PCR) assays (Fig. 1C). To determine the effect of MeCP2 knockdown on the activation status of AKT, the well-known downstream of PTEN, we compared the phosphorylation level of AKT at Thr308 in primary cultured neurons transfected with lentivirus expressing GFP, PTEN RNAi (for PTEN knockdown), and MeCP2 RNAi. Remarkably, AKT phosphorylation was consistently up-regulated by PTEN and MeCP2 RNAi, compared with control cells (Fig. 1D). These results suggest that knockdown of MeCP2 photocopy the alternations in signaling pathway similar with knockdown of PTEN.

### miR-137 is intermediate in MeCP2 regulation of PTEN

**Figure 2:**
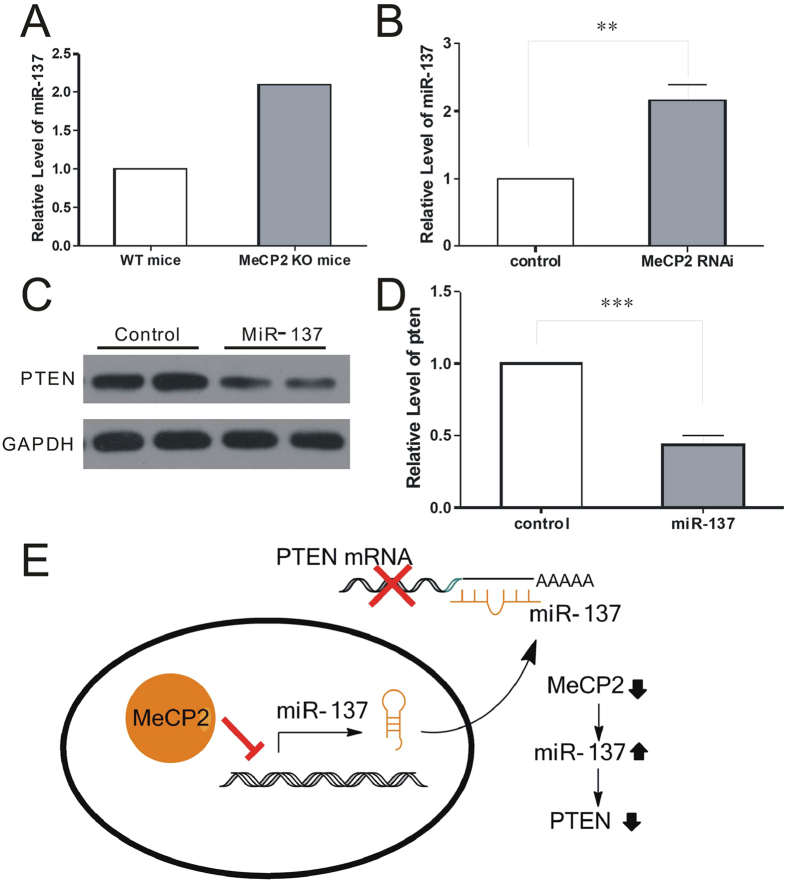
MeCP2 regulates PTEN expression via miR-137. **(A)** Deep-sequencing data showed a > 2.5-fold increase in miR-132 expression in MeCP2 KO mice. **(B)** miR-137 expression was upregulated > 2-fold in MeCP2 RNAi neurons. **(C)** PTEN protein expression was down-regulated in neurons transduced with miR-137 mimic. **(D)** PTEN protein levels in (C) were quantified using Image J. **(E)** Signaling pathway for MeCP2 regulating PTEN expression in neurons.

It is previous reported that MeCP2 represses miR-137 expression in neural stem cells^15^. Indeed, we found that miR-137 was up-regulated for over 2.1 fold in *Mecp2*-knockout mouse cortical neurons, as demonstrated by Solexa-based RNA sequencing (RNA-seq) (Fig. 2A). We also confirmed that pri-miR-137 levels were increased in primary cultured neurons transfected with lentivirus expressing GFP or MeCP2 RNAi compared with control neurons (Fig. 2B). To determine if miR-137 regulated the expression of PTEN, we transduced primary cultured neurons with miR-137 mimic oligonucleotides. Overexpression of miR-137 mimic reduced PTEN expression by about 56% (Figs 2C,D). These results suggest the existence of a regulatory cascade from MeCP2, miR-137, to PTEN (Fig. 2E).

### Pten knockdown suppressed MeCP2 expression

**Figure 3:**
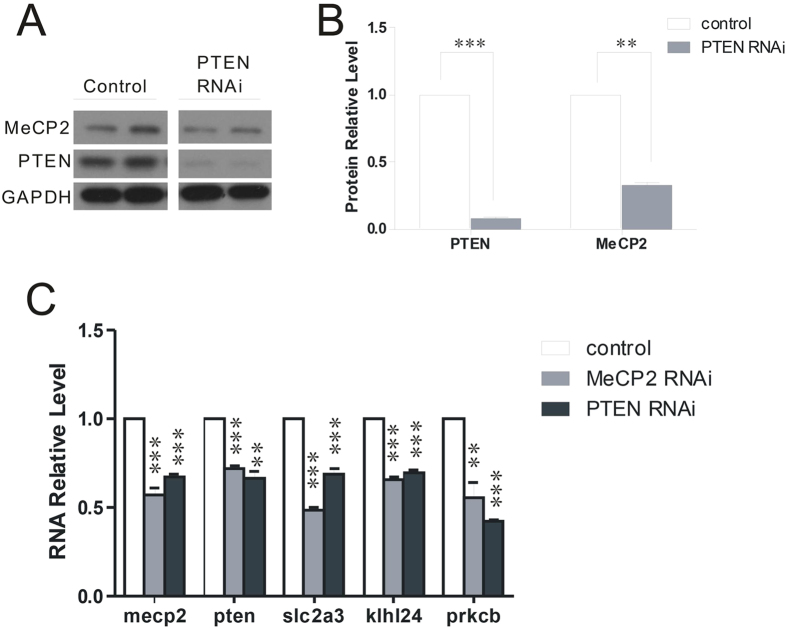
MeCP2 is regulated by PTEN. **(A)** Protein levels of PTEN and MeCP2 were downregulated in cultured neurons infected with lentivirus harboring PTEN RNAi (for PTEN knockdown), as demonstrated by Western blotting. **(B)** MeCP2 and PTEN protein levels in (**A**) were quantified using Image J. **(C)** Relative quantities of *Mecp2, Pten, Slc2a3, Klhl24,* and *Prkcb* showed that PTEN downregulated MeCP2 target genes.

Next in an experiment of knocking down PTEN with short hairpin RNA, we surprisingly found that reducing the expression of *Pten* resulted in a marked decrease in expression of MeCP2 (0.33 ± 0.03 vs. 1.00; P < 0.01) (Figs 1A and 3A,B). Similarly, *Mecp2* mRNA expression was downregulated by PTEN RNAi (0.67 ± 0.02 vs. 1.00; P < 0.001) (Fig. 3A). We tested the regulatory effect of PTEN on the MeCP2-target genes *Slc2a3, Klhl24,* and *Prkcb*, which were decreased > 2-fold in *Mecp2-*knockdown cultured neurons, as demonstrated by RNA-seq (data not shown). *Slc2a3, Klhl24,* and *Prkcb* were significantly reduced by *Pten* knockdown, as confirmed by real-time PCR (Fig. 3C), suggesting that knockdown of MeCP2 and knockdown of PTEN may share common downstream targets.

### PTEN regulated CREB phosphorylation and miR-132

**Figure 4:**
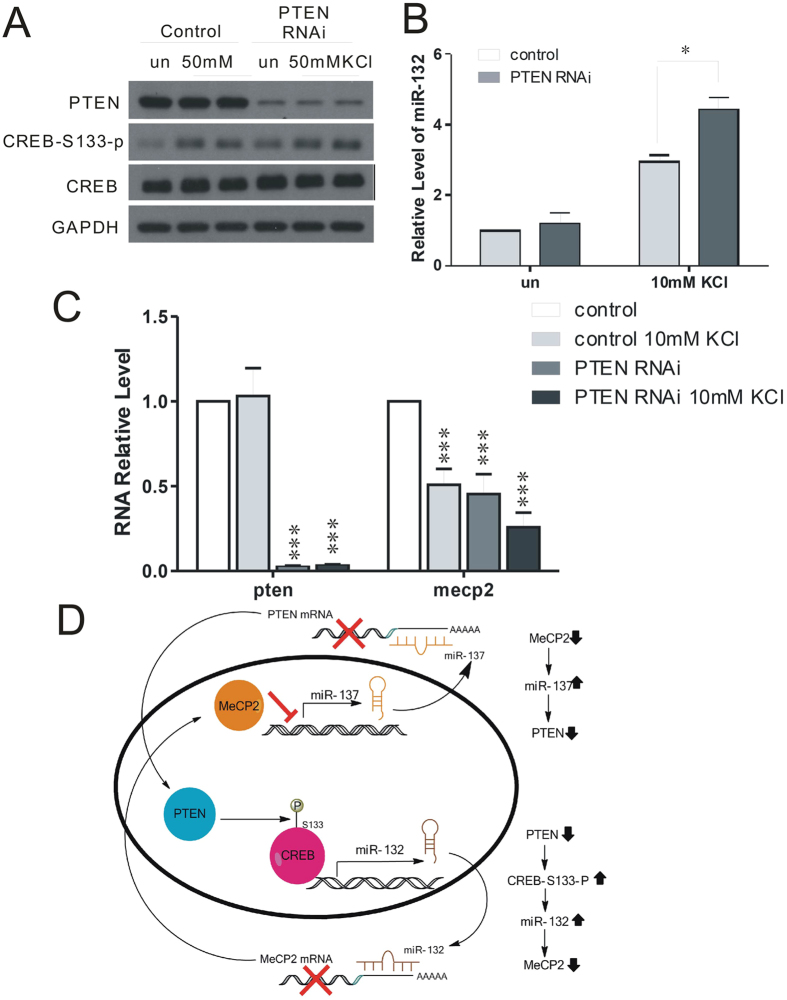
PTEN regulates MeCP2 via CREB-mediated miR-132 expression. **(A)** Western blotting analysis of CREB phosphorylation in PTEN RNAi neurons with 50 mM KCl stimulation for 30 min. **(B)** miR-132 levels were increased in Pten-knockdown neurons stimulated with 10 mM KCl for 2 h, as shown by quantitative real-time PCR analysis. **(C)** *Mecp2* expression was significantly decreased in PTEN RNAi neurons with 10 mM KCl stimulation. **(D)** Signaling pathway for reciprocal regulation between MeCP2 and PTEN.

As PTEN has been found to regulate the de-phosphorylation of CREB, which plays a critical role in regulating microRNA-132^23^. To investigate the molecular mechanism by which PTEN regulates the expression of MeCP2, we examined CREB phosphorylation at Ser133 in primary cultured neurons with or without PTEN upon KCl stimulation. As shown in Fig. 4A, PTEN deletion together with 50 mM KCl stimulation resulted in a significant increase in CREB phosphorylation. MeCP2 is reportedly regulated by the CREB-induced miRNA, miR-132^24^,^25^. To determine if the CREB-induced increase in miR-132 may result from reduced PTEN levels, we examined miR-132 levels in PTEN RNAi neurons stimulated with 10 mM KCl, compared with KCl-stimulated control neurons. miR-132 levels were 50% higher in PTEN RNAi compared with control neurons (2.95 ± 0.175 vs. 4.43 ± 0.34; P < 0.05) (Fig. 4B).

Finally, we transfected mouse primary cortical neurons with GFP and PTEN RNAi followed by depolarization with 10 mM KCl for 2 h. *Mecp2* expression was dramatically suppressed by KCl-induced PTEN repression (Fig. 4C). Overall, these results indicate that PTEN knockdown suppresses expression of MeCP2 and its target genes in neurons.

Taken together, we therefore proposed that PTEN dephosphorylated CREB at Ser133 and repressed miR-132, resulting in the reciprocal regulation of MeCP2. Thus, knockdown PTEN leads to increased phosphorylation level of CREB Ser-133 site, which in turn promotes expression of miR-132 and then decreases expression of MeCP2 (Fig. 4D).

## Discussion

Emerging evidence implicates PTEN and MeCP2 as critical regulatory factors in various aspects of the central nervous system. However, the interrelationship between the two genes has proved elusive. Our results suggest that two specific miRNAs act as intermediates in the reciprocal regulation of PTEN and MeCP2. We showed that inhibition of MeCP2 affected the overexpression of miR-137 and decreased PTEN expression, while overexpression of miR-137 could also inhibit the expression of PTEN, suggesting that miR-137 is critical to the regulation of PTEN by MeCP2. We also demonstrated that PTEN regulated MeCP2 via the transcription factor CREB, and another specific miRNA, miR-132. Previous studies found that CREB acted as a protein substrate of PTEN phosphatase in the nucleus^23^, and miR-132 inhibitors largely blocked the effects of CREB on dendrite maturation^24^. MeCP2 translation has also been reportedly regulated by miR-132, and blocking miR-132-mediated repression increased MeCP2 levels in cultured neurons, and loss of MeCP2 reduced miR-132 levels *in vivo*^25^. Our results indicated that CREB may be phosphorylated by KCl-induced inhibition of PTEN. In addition, pri-miR-132 levels were increased by KCl stimulation in PTEN RNAi neurons. These results suggest that CREB and miR-132 may be involved in the regulation of MeCP2 by PTEN.

In conclusion, we demonstrated that MeCP2 regulates PTEN expression via the specific miRNA, miR-137. Conversely, PTEN can indirectly regulate MeCP2 via CREB and miR-132. Demonstration of this reciprocal regulation between PTEN and MeCP2 may provide the basis for more detailed studies of the mechanisms of these critical genes.

## Methods

### Primary neuron cultures and transduction, Mice

E15.5 mouse cortical neurons were cultured and transduced with Amaxa Nucleofector or transfected with lentiviruses (10^8-9^ vg/ml) (packaged by Neuron Biotech, Shanghai). Cells were collected at 5 days. Experiments were performed for over three times. All experimental procedures were complied with the guidelines and under the approval of the Animal Care and Use Committee of the Shanghai Institute for Biological Science of the Chinese Academy of Sciences.

### Plasmid construction

The RNAi targeting sequences were as follows: PTEN shRNA: 5′- AGA CAA GGC CAA CCG ATA C-3′; MeCP2 shRNA: 5′- AAG TCA GAA GAC CAG GAT C-3′.

### Western blotting

Protein samples were separated by sodium dodecyl sulfate-polyacrylamide gel electrophoresis and transferred to polyvinylidene fluoride membranes (Millipore, Billerica, MA, USA). Membranes were processed according to the ECL Western Blotting Protocol. α-MeCP2 (Cell Signaling Technology, Boston, MA, USA, 1:1000 dilution), α-PTEN (Cell Signaling Technology, 1:1000 dilution), α-CREB (Cell Signaling Technology, 1:1000 dilution), α-p-CREB (Cell Signaling Technology, 1:1000 dilution), and control α-glyceraldehyde phosphate dehydrogenase (Abcam, Cambridge, MA, USA, 1:5000 dilution) were used as primary antibodies. All Western blot quantifications were performed using Image J software.

### Real-time PCR relative quantification

Total RNA from cultured neurons was extracted using TRIzol Reagent (Invitrogen, Carlsbad, CA, USA). Reverse transcription was carried out with Reverse Transcriptase M-MLV (RNase H-free) (Takara Bio, Otsu, Japan) and quantitative PCR was carried out using a Rotor-Gene Q (Qiagen, Valencia, CA). Primer sequences were as follows: *Pten*-forward: 5′-TGG ATT CGA CTT AGA CTT GAC CT-3′, *Pten*-reverse: 5′-TGG CGG TGT CAT AAT GTC TCT-3′, *Mecp2*-forward: 5′-ACA GCG CTC CAT TAT C-3′, *Mecp2*-reverse: 5′- CCC AGT TAC CGT GAA GTC AAA A-3′, *Slc2a3*-forward: 5′-ATG GGG ACA ACG AAG GTG AC-3′, *Slc2a3*-reverse: 5′- CAG GTG CAT TGA CTC CAG-3′, *Klhl24*-forward: 5′- GGA TCT TGG GGT GCG TGA TT-3′, *Klhl24*-reverse: 5′- GGA CAG CTC GAT GGC ATG G-3′, *Prkcb*-forward: 5′- ATG AGT TCG TCA CGT TCT CCT-3′, *Prkcb*-reverse: 5′- CCA TAC AGC GAT CCA CAG-3′, pri-miR-132-forward: 5′-ACC GTG GCT TTC GAT TGT TA-3′, pri-miR-132-reverse: 5′-GGC GAC CAT GGC TGT AGA CT-3′, and pri-miR-137-forward: 5′-ACT CTC TTC GGT GAC GGG TA-3′, pri-miR-137-reverse: 5′-CGC TGG TAC TCT CCT CGA CT-3′.

### RNA-seq Deep Sequencing

One P30 MeCP2 KO mouse and one P30 wide type mouse cortex RNA samples were harvested as described by Kwak *et al*. (2009) Both samples were subjectedto Solexa of BGI Shenzhen.

### Statistical analysis

All results are expressed as mean ± SEM and were analyzed using Stata v. 10 software (Stata Corporation, College Station, TX USA). Results were compared using two-sample *t*-tests with equal variance. A P value < 0.05 was considered statistically significant.

## Additional Information

**How to cite this article**: Lyu, J.-W. *et al*. Reciprocal regulation of autism-related genes MeCP2 and PTEN via microRNAs. *Sci. Rep.* **6**, 20392; doi: 10.1038/srep20392 (2016).
